# Importance of Population-Based Cancer Risk Information in the Care of Patients With Rare Genetic Disorders

**DOI:** 10.1200/JCO.21.02251

**Published:** 2021-11-18

**Authors:** Sharon E. Plon

**Affiliations:** ^1^ Baylor College of Medicine and Texas Children's Hospital, Houston, TX

One of the first things that a geneticist is asked by family members and other clinicians caring for a child diagnosed with a rare chromosomal instability disorder is to quantify the child's cancer risk. It is simply not enough to say that they are at high risk of cancer. Precise cancer risk estimates are critical for the development of cancer surveillance and prevention guidelines. For example, in the series of articles derived from the American Association of Cancer Research Cancer Predisposition Workshop, the decision was made to not recommend cancer surveillance for malignancies with a less than one percent cancer risk per year (during the pediatric period).^1^ The cancer genetics literature contains many articles describing cancer incidence in case series or registries of patients with a genetic disorder, but this work is inherently biased as patients without cancer may not seek care in a medical center or even be diagnosed with the genetic disorder in question. Thus, it is difficult to use the cancer risk estimates from these types of studies when advising families or developing guidelines.

**THE TAKEAWAY**

In the article that accompanies this editorial, Dutzmann et al^2^ leverage both national genetic diagnostic and childhood cancer registry data to estimate the risk of cancer in the pediatric age range for individuals with these two rare chromosome instability disorders. The high cancer risk and the specific pattern of malignancies reported should enable clinicians to develop more appropriate cancer surveillance and treatment regimens with the goal of improving survival and diminishing morbidity.

The article that accompanies this editorial, Dutzmann et al,^2^ circumvents these biases by using two unique nationwide resources in Germany for children with Fanconi anemia (FA) and ataxia telangiectasia (AT). The analytical approach was the same for both disorders. The genetic diagnoses were based on analysis of data from nationwide reference laboratories—at the University of Würzburg and Hannover Medical School—responsible for confirming this diagnosis in any patient suspected of these disorders in Germany between the period of 1973 and 2020. The second nationwide resource was the German Childhood Cancer Registry, which has been responsible since 1980 to monitor incident cases of malignancies up to age 15 years (until 2008) and subsequently up to age 17 years. Like most cancer registries, nonmalignant brain tumors, for example, low-grade gliomas, were also included. The linkage of these two national data sets provides the primary results for this article without the biases previously described. This type of registry linkage is particularly powerful when dealing with rare disorders. Another example of the use of nationwide diagnostic cohorts for rare disorders is the analysis of meningioma surgery outcomes for patients with neurofibromatosis type 2 in France.^3^ These types of studies are difficult in the United States, given the lack of national reference laboratories and health care systems. However, Lupo et al^4^ recently linked statewide birth defect and cancer registries in the United States to determine the risk of cancer in children with specific birth defects.

It is disappointing that the investigators did not have access to the same type of cancer registry data for adults with these disorders in Germany, particularly given the increasing use of hematopoietic stem-cell transplant (HSCT) for FA. These individuals are living longer into adulthood and are demonstrating increased risk of malignancies such as head and neck cancer. Two recent reports of survivors of HSCT for FA demonstrated that 40%-70% (depending on the length of follow-up) of individuals developed squamous cell carcinoma of the head and neck region.^5,6^ The situation with AT and adult-onset cancers is more complex as many patients with AT do not survive far into adulthood, given the combined risk of malignancy, immunodeficiency, and severe neurologic disability, resulting in tragic losses because of pulmonary failure during adolescence and young adulthood.^7,8^

Dramatically different approaches for identifying every individual with a genetic condition in a geographic region have more recently involved genome-scale sequencing such as the study of individuals with *DICER1* pathogenic variants in the Geisinger Health population^9^ or conversely using highly specific and sensitive clinical features, for example, the astounding study where investigators systematically performed physical examinations on 152,819 six-year-old children in six German states using the neurofibromatosis type 1 diagnostic criteria to identify all individuals with neurofibromatosis type 1.^10^ These studies, like that by Dutzmann et al,^2^ share the goal of studying unbiased populations of individuals with rare genetic disorders to inform the most appropriate cancer prevention and surveillance regimens.

This study of patients with FA and AT was able to incorporate patient data starting in the 1970s—long before DNA diagnostics were developed—because of the availability of cell-based diagnostic assays. There are multiple rare autosomal and X-linked recessive disorders where the deficiency state of the patient's cells results in unique cellular phenotypes that are assayed in reference laboratories. As described in this article, hypersensitivity of cells in response to mitomycin C or diepoxybutane is diagnostic of FA and cellular sensitivity to ionizing radiation for AT. Other disorders with cellular phenotypes frequently used for diagnosis^11^ include the following: dyskeratosis congenita (telomere length), xeroderma pigmentosum (cellular sensitivity to ultraviolet radiation), and Bloom syndrome (increased sister chromatid exchange). Although there is an increasing development of sequencing-based assays for patients suspected of Mendelian disorders, one should not ignore the high sensitivity and specificity of these cellular assays, particularly for disorders like FA where identification of the underlying genetic causes has been ongoing for decades. Twenty-two FA genes have been identified to date with the publication of *FANCW*^12^ as recent as 2017.

As expected from previous literature, the overall childhood cancer risks are significantly increased compared with children in the German population with the standardized incidence ratio of 39 (95% CI, 26 to 56) for FA and 56 (95% CI, 33 to 88) for AT. Although useful information, it can be hard for parents and clinicians to grasp what such a high standardized incidence ratio means regarding the absolute cancer risk in an individual patient. The study^2^ provides some of the first population-based estimates of cancer risk to age 17 years, with 11% for patients with FA and 14% for patients with AT. In addition, the study^2^ provides the specific tumor type(s) each patient with FA or AT developed. What is quite striking is the prevalence of hematopoietic malignancies for both disorders. Although this was clearly recognized previously, the large number of individual case reports describing patients with FA and solid tumors has made it difficult to assess what is the relative risk of liquid versus solid tumors in this disorder. Of the 33 malignancies in patients with FA, 25 were hematopoietic cancers, and for patients with AT, it was 18 of 19 malignancies, with the majority being lymphoma.

A recent review^13^ of the many factors to consider when deciding on the timing of HSCT for acute myeloid leukemia (AML) predisposition syndromes noted that for those conditions where myelodysplasia develops before AML, HSCT can sometimes be delayed until early features of myelodysplastic syndrome (MDS) develop. Those authors also note that the likelihood of AML diagnosis (or penetrance) is a critical feature in weighing the potential benefit of pre-emptive HSCT, given the risks associated with the procedure. In the study by Dutzmann et al,^2^ multiple FA patients presented with MDS before AML and the overall penetrance for any malignancy was 11% (compared with much higher AML penetrance disorders, eg, familial AML with *CEPBA* mutations). Thus, HSCT may be delayed until there are clear indications of disease progression, such as increased transfusion requirements or karyotypic changes on bone marrow evaluation, detected through surveillance studies (see recommendations from Fanconi Anemia Research Foundation^14^). For AT, there were no patients with AML among the cohort of 160 patients and only 3 with ALL, thus making pre-emptive HSCT as a leukemia preventive measure unfavorable, which is consistent with the recommendations of the Ataxia Telangiectasia Children's Project.^15^

These relative risks of tumor types are particularly important when updating surveillance guidelines. As described in the work by Porter et al,^16^ screening individuals at increased risk of hematopoietic malignancies is very controversial and patterns differ worldwide. As above, disorders like FA with myelodysplasia before AML may be most appropriate for more intensive hematologic and bone marrow surveillance.

Patients diagnosed with FA resulting from pathogenic variants in the genes encoding the FA core complex have MDS or AML as their first malignancy, with a few carcinomas (not otherwise described) as second malignancies. By contrast, those FA genes that encode proteins more closely related to RAD51-mediated DNA repair mechanisms, for example, *BRCA2* or *PALB2*, have a much higher risk of diverse solid tumors. Thus, patients diagnosed with FA based solely on diepoxybutane or mitomycin C breakage assay should have subsequent DNA analysis to identify the specific FA subtype to predict cancer risk more accurately. Those patients with individually rare, non-FA core complex subtypes might be considered for solid tumor screening regimens similar to those recommended for Li Fraumeni syndrome^17^ or constitutional mismatch repair deficiency syndrome.^18^ Conversely, the very low risk of solid tumors, outside of lymphoma, in patients with AT would argue that surveillance modalities such as region-specific or whole-body magnetic resonance imaging are not warranted.
